# The Potential of Nuclear Pore Complexes in Cancer Therapy

**DOI:** 10.3390/molecules29204832

**Published:** 2024-10-12

**Authors:** Hanna Zaitsava, Martyna Gachowska, Elżbieta Bartoszewska, Alicja Kmiecik, Julita Kulbacka

**Affiliations:** 1 Students’ Group of Cancer Cell Biology, Faculty of Medicine, Wroclaw Medical University, Mikulicza-Radeckiego 5, 50-345 Wroclaw, Poland; hanna.zaitsava@student.umw.edu.pl (H.Z.); elzbieta.bartoszewska@student.umw.edu.pl (E.B.); 2 Department of Histology and Embryology, Wroclaw Medical University, 6a Chałubińskiego St., 50-368 Wroclaw, Poland; alicja.kmiecik@umw.edu.pl; 3 Department of Molecular and Cellular Biology, Faculty of Pharmacy, Wroclaw Medical University, Borowska 211A, 50-556 Wroclaw, Poland; 4 Department of Immunology and Bioelectrochemistry, State Research Institute Centre for Innovative Medicine, Santariškių g. 5, LT-08406 Vilnius, Lithuania

**Keywords:** nuclear pore complex, nanopore, nuclear transport, nanotherapy

## Abstract

Nuclear pore complexes (NPCs) play a critical role in regulating transport-dependent gene expression, influencing various stages of cancer development and progression. Dysregulation of nucleocytoplasmic transport has profound implications, particularly in the context of cancer-associated protein mislocalization. This review provides specific information about the relationship between nuclear pore complexes, key regulatory proteins, and their impact on cancer biology. Highlighting the influence of tumor-suppressor proteins as well as the potential of gold nanoparticles and intelligent nanosystems in cancer treatment, their role in inhibiting cell invasion is examined. This article concludes with the clinical implications of nuclear export inhibitors, particularly XPO1, as a therapeutic target in various cancers, with selective inhibitors of nuclear export compounds demonstrating efficacy in both hematological and solid malignancies. The review aims to explore the role of NPCs in cancer biology, focusing on their influence on gene expression, cancer progression, protein mislocalization, and the potential of targeted therapies such as nuclear export inhibitors and intelligent nanosystems in cancer treatment. Despite their significance and the number of research studies, the direct role of NPCs in carcinogenesis remains incompletely understood.

## 1. Introduction

Nuclear pore complexes (NPCs) represent intricate structures embedded within the nuclear envelope, serving as paramount conduits for orchestrating molecular exchange between the nucleoplasm and the cytoplasm in eukaryotic cells [1]. These complexes are pivotal in transport-dependent gene expression, controlling access to transcription factors. This regulatory function ensures the fidelity of signal transduction pathways, thereby averting the untoward activation of cellular processes [2]. Even subtle perturbations in the composition or functional dynamics of these pore complexes can precipitate a cascade of events leading to the misregulation of cellular growth and the aberrant subcellular localization of proteins [3].

The consequential impact of dysregulated nucleocytoplasmic transport profoundly affects overall cell function and is an important factor in the etiology of various diseases, including cancer. NPCs can be a strategic tool for cell signaling regulation, as it has been noted that they create specific channels for nucleocytoplasmic exchange [4]. Dysregulated nucleocytoplasmic transport develops from a variety of factors beyond defects in nuclear pore complexes (NPCs), involving complex regulatory processes. Misregulated signal transduction pathways, such as those involving mitogen-activated protein kinases (MAPKs), can alter the phosphorylation state of proteins, disrupting their interaction with transport receptors and leading to improper nuclear import or export. Post-translational modifications (PTMs), including phosphorylation, ubiquitination, acetylation, and sumoylation, can affect nuclear localization or export signals, preventing proper recognition by transport receptors. Additionally, amino acid substitutions in key regulatory proteins may disrupt nuclear localization or export sequences, resulting in mislocalization. Furthermore, dysregulation of transport receptors like CRM1/exportin 1, through overexpression or mutation, can promote the mislocalization of tumor suppressors or oncogenic factors, shifting the balance of critical proteins in favor of cancer progression. Even structural changes to the nuclear envelope can affect the overall efficiency of nucleocytoplasmic transport, further contributing to the disruption of protein localization in cancer cells. Together, these factors highlight the complexity of transport dysregulation and its role in disease [5]. Thus, comprehending nuclear pore complexes’ intricate structure and nuanced functionality is paramount in unraveling their multifaceted roles within cellular processes. Additionally, understanding NPCs holds promise for developing potential therapeutic strategies to rectify dysregulations in nucleocytoplasmic transport and mitigate the associated pathological consequences. The convergence of structural insights and functional delineation is instrumental in decoding the enigmatic contributions of NPCs to cellular homeostasis and pathological states [6].

### 1.1. NPCs’ Structure

**Figure 1:**
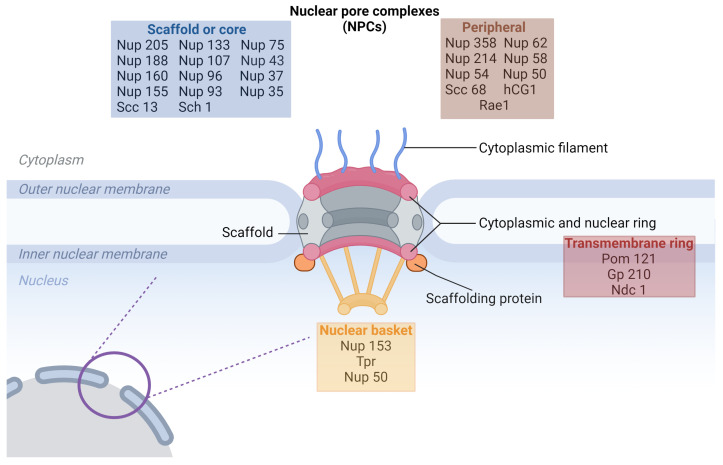
Schematic illustration of the nuclear pore complex (NPC) structure highlighting the relative location of different nucleoporin (Nup) classes. The NPC is embedded within the nuclear envelope, composed of the outer and inner nuclear membranes. The scaffold or core Nups (blue box) form the central framework of the NPC and include proteins such as Nup205, Nup160, and Sec13, which span both the cytoplasmic and nuclear sides. The peripheral Nups (brown box) are associated with the cytoplasmic filaments and nuclear basket structures, including Nup358, Nup214, and Rae1. The transmembrane ring Nups (red box), such as Pom121, Gp210, and Ndc1, are embedded within the nuclear membrane, anchoring the NPC in the nuclear envelope. The nuclear basket (yellow box), containing proteins like Nup153 and Tpr, is located on the nucleoplasmic side, contributing to transport regulation. This structure enables the NPC to mediate nucleocytoplasmic transport effectively [20].

NPCs form extensive protein complexes within eukaryotic cells, penetrating both the inner and outer membranes of the nuclear envelope (NE). These gate-like structures facilitate regular molecular exchange between the nucleus and cytoplasm (Figure 1) [7,8].

Cryo-electron microscopy has unveiled that the NPC assembly adopts an eight-fold symmetrical structure, enveloping a central transport channel. This intricate ensemble consists of approximately 30 distinct nucleoporins (Nups), appearing in multiple copies and organized into various subcomplexes that regulate the nucleoplasmic trafficking of macromolecules. Nups allow the maintenance of genome integrity and mitotic progression and are crucial for gene expression regulation [9,10,11]. The NPC plays a crucial role in regulating the cell cycle, influencing processes such as proliferation or apoptosis in both normal and malignant cells. Particularly in malignant cells, the transport through the NPC is exploited to stimulate tumor growth and evade apoptotic pathways, underscoring the significance of nucleocytoplasmic transport in the context of cancer biology [12,13,14].

Nups within the NPC can be categorized into three groups (Table 1). The scaffold Nups, such as Nup107-160 and Nup93-205, form the core of the NPC. Intriguingly, Menon et al. demonstrated that these subcomplexes may act as transcription activators. The second group is the transmembrane group, including NupsPOM121, NDC1, and gp210, which remains tethered to the NE. The third group, comprising peripheral Nups, plays a crucial role in mediating the selectivity and permeability of the nuclear pore, contributing to the functional integrity of nucleocytoplasmic transport [15,16,17,18].

There is a possibility to distinguish another group of Nups, responsible for anchoring the NPC to the NE: those containing a phenylalanine–glycine (FG) domain—including Nup62, Nup54, Nup45, and Nup58. These FG-domain-containing nucleoporins provide crucial interaction domains for the regulated transport of molecules across the NPC. Mutations or changes in the expression levels of these nucleoporins may weaken the NPC’s ability to form an effective diffusion barrier, leading to improper or uncontrolled transport of molecules between the nucleus and cytoplasm. This could result in the mislocalization of key regulatory proteins, such as transcription factors, and contribute to diseases like cancer by disrupting the delicate balance of nuclear-cytoplasmic trafficking. Additionally, dysregulation in these FG nucleoporins could impair their role in anchoring the NPC to the nuclear envelope, further destabilizing NPC function and contributing to broader nucleocytoplasmic transport defects. Notably, this structural arrangement serves as a primary diffusion barrier within the NPC, contributing significantly to the controlled and selective passage of molecules between the nucleus and cytoplasm [14,19].

The Nup94 complex, positioned on the outer part of the NPC facing the cytoplasm, comprises Nup120, Nup85, Seh1, Sec13, Nup145c, and Nup84. This complex contributes to the structural integrity of the outer face of the NPC and participates in the regulation of molecular traffic. Furthermore, Nup133 plays a pivotal role in ensuring the proper distribution of individual NPCs around the nuclear envelope [14,21].

According to a study by D’Angelo et al., the organization of Nups inside cells differs in dividing and terminally differentiated cells. This observation underscores the dynamic nature of the NPC’s composition and organization, suggesting that the intricate arrangement of Nups is responsive to the specific functional requirements associated with different cellular states, such as cell division and terminal differentiation [22].

Nups collectively shape the tripartite structure of the NPC: the cytoplasmic ring, nuclear ring, and spoke complex. The spoke complex consists of an eight-fold symmetric central framework extending between two rings. Within this complex, a central pore facilitates the macromolecule’s regulated passage between the nucleus and cytoplasm. The cytoplasmic and nuclear rings function as anchors between the NPC and the nuclear envelope. The cytoplasmic ring exhibits loose ends on one side, while the nuclear ring, featuring eight nuclear filaments, forms a nuclear pore basket. This basket plays a pivotal role in processing and sorting molecules during their transition through the NPC, serving as docking sites for nuclear transport events. Notably, the location of the translocated promoter region (TPR) within these rings is essential for interactions with karyopherins involved in classical nuclear import and export processes [14,23].

**Table 1:** NPC proteins and their function.

Protein	Function	Reference
**scaffold**		
Nup93/Nup205 complex (Nup93, Nup205, Nup188, Nup155, Nup35),	forming of central pore	[11]
Nup107/Nup160 complex (Nup107, Nup160 Nup133, Nup 96, Nup43, Nup37, Nup 85, Sec13, Seh1, Elys)	forming of cytoplasmic and nuclear ring	[11]
**transmembrane **		
Pom121, Gp210, Ndc1	linking of NPC to the nuclear envelope	[11]
**peripheral **		
Nup358/RanBP2, Nup214, Nup 88	forming of cytoplasmic filaments	[11]
Nup153, Tpr	forming of nuclear basket	[11]
Nup98, Rae1	shuttling transport factors	[24]
**FG-domain-anchoring Nups**		
Nup62 complex (Nup62, Nup58, Nup54, Nup45)	selective permeability barrier regulation of receptor-mediated translocation	[11]

### 1.2. NPCs’ Function

Eukaryotic cells encapsulate their genetic material within the nucleus, which is why there is a need for efficient communication between the nucleus and cytoplasm in order to regulate the cell cycle and cellular viability. NPCs are crucial in facilitating nucleocytoplasmic transport through both passive and facilitated diffusion mechanisms. This dynamic process ensures the controlled exchange of macromolecules, including proteins and RNA, between the nucleus and cytoplasm, thus contributing to fundamental cellular functions and maintaining cellular homeostasis [25].

Smaller molecules, such as ions or water or macromolecules below 40 kDa, can freely diffuse through the NPC, while larger molecules follow a more intricate pathway. The transport selectivity relies on two recognition mechanisms. The first involves binding to transport proteins directly or through adaptor proteins, and the second involves direct interaction with the FG nucleoporins. Karyopherins, including importins and exportins, serve as receptor proteins responsible for facilitating the passage of large cargoes through NPCs, overcoming apparent concentration gradients. This process is orchestrated through the recognition of the nuclear import signals and nuclear export signals carried by the respective cargoes [12,26].

**Figure 2:**
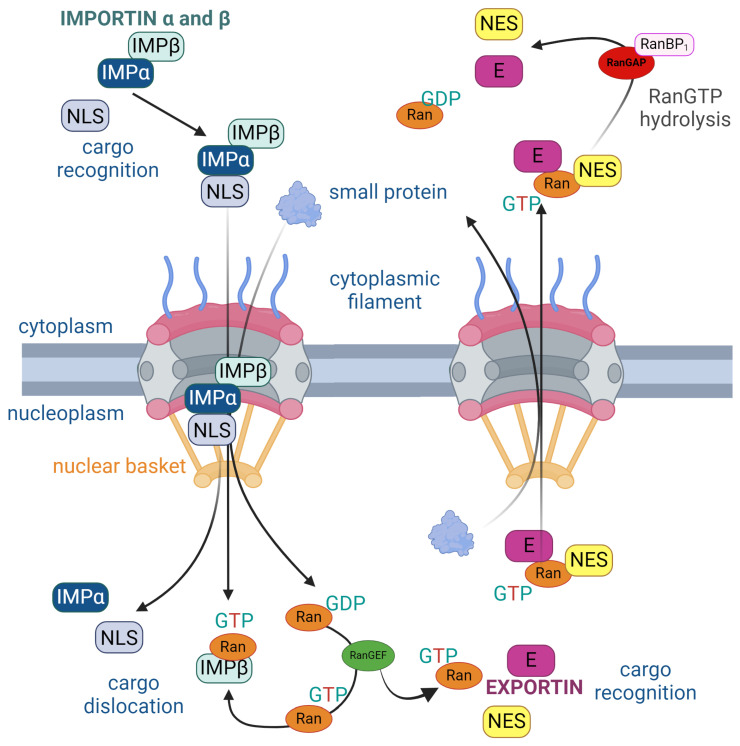
Structure of the nuclear pore complex (NPC) illustrating the unidirectional nuclear import and export of proteins regulated by the RanGTP cycle. On the left, nuclear import is facilitated by importins (IMP), which recognizes cargo with a nuclear localization signal (NLS) in the cytoplasm. Importins α and β bind to the NLS and carry the cargo through the NPC into the nucleus. Inside the nucleus, RanGTP binds to importin β, causing cargo dislocation and its release into the nucleoplasm. On the right, nuclear export involves exportin (E), which recognizes cargo containing a nuclear export signal (NES). Exportin binds to both the NES and RanGTP to facilitate export through the NPC. Once in the cytoplasm, RanGTP is hydrolyzed to RanGDP by RanGAP and RanBP1, causing the release of the cargo into the cytoplasm. The cycle is maintained by RanGEF, which facilitates the conversion of RanGDP to RanGTP inside the nucleus. The arrows represent the direction of protein movement, the progression of complex formation and disassembly, and the key interactions in nuclear transport [28].

Each cargo is characterized by nuclear localization signals (NLS) or nuclear export signals (NES), enabling their binding to karyopherins. This interaction is essential for the engagement of the NPC. Without this recognition, the NPC remains closed to larger cargoes (>40 kDa). The classical import/export pathways involve the formation of an importin β/importin α NLS–cargo complex in the cytoplasm or a CRM1-GTP-bound (CRM1-nuclear export factor Chromosomal Region Maintenance 1, also known as XPO-1) RAN–NES–cargo complex. This strategic coordination ensures the effective translocation of the cargo across the nuclear envelope through the NPC (Figure 2) [27].

Subsequent to the formation of the importin β/importin α–NLS cargo complex or CRM1-GTP-bound RAN–NES–cargo complex, karyopherins dock onto the TPR of the nuclear basket. The directionality of this passage is guided by the two nucleotide states of the GTPase Ran. Notably, RanGTP concentration is higher in the nucleus. Exportins exhibit the ability to bind to specific cargo in the presence of RanGTP, facilitating the passage of the molecule to the cytoplasm. Subsequently, GTP hydrolysis leads to the breakdown of the export complex, completing the nucleocytoplasmic transport cycle [23,29,30].

The protein–cargo complex is capable of bidirectional movement through the NPC. Throughout the transport cycle, thermal diffusion is facilitated by the interaction with GTP hydrolysis, generating a thermal standpoint and causing conformational changes in the FG nucleoporins. These changes enable hydrophobic interactions between FG motifs and hydrophobic pockets of the transported protein. The NFT2 transport protein orchestrates the reverse import of RanGDP and is converted back to RanGTP by nucleotide exchange, which is ready to reinitiate the transport cycle. In summary, the direction of transport is governed by the energy derived from irreversible GTP hydrolysis in the cytoplasm, and it is sustained by the localization gradient of RanGTP/RanGDP [31,32].

According to Capelson et al., NPCs extend beyond their classical role in nuclear transport. NPCs have been found to have additional functions in chromatin organization and gene regulation that operate independently of nuclear transport. NPCs may directly influence gene expression through physical interactions between Nups and the nuclear genome. This involvement can impact both the organization of chromatin within the nucleus and the transcriptional status of genes that are physically associated with pore components. This expanded role highlights the multifaceted contributions of NPCs to cellular processes beyond their canonical function in nucleocytoplasmic transport [20].

The dysregulation of nucleocytoplasmic transport has profound implications for various steps of cancer development and progression. This process plays a crucial role in the regulation of gene expression, cell cycle dynamics, and proliferation. It has been established that malignant cells exploit nucleocytoplasmic transport to stimulate tumor growth and evade apoptotic mechanisms. They evade anti-neoplastic therapies via aberrant localization of tumor-suppressor proteins [6]. The aberrant functioning of the NPC can thus contribute to the hallmarks of cancer, underscoring the importance of understanding and targeting nucleocytoplasmic transport mechanisms in the context of cancer biology [13,14].

The process of nucleocytoplasmic transport through the NPC is intricately coordinated. Specific transcription factors, when activated, follow highly regulated transcriptional programs that play a crucial role in determining cell fate as they traverse through the NPC. This coordination underscores the significance of precise molecular interactions and signaling events at the NPC in influencing gene expression and ultimately shaping cellular functions and fate [6,33]. The nuclear export mediator XPO1, also known as CRM1, has gained attention as a critical target in cancer therapy due to its overactivity in various malignancies, including ovarian carcinoma, glioma, osteosarcoma, pancreatic, and cervical cancers. XPO1 is essential for regulating nucleocytoplasmic transport by exporting proteins that contain nuclear export signals (NES), many of which are key oncogenes and tumor suppressors. Gravina et al. emphasize that XPO1 influences cancer progression through multiple pathways: (i) controlling the subcellular localization of NES-containing proteins, (ii) regulating mitotic apparatus and chromosome segregation, and (iii) maintaining nuclear and chromosomal structures. XPO1 achieves these functions by interacting with nucleoporins, such as Nup214 and Nup88, which are crucial for docking and transporting cargo across the nuclear pore complex (NPC). The dysregulation of XPO1 disrupts the delicate balance between oncogenes and tumor suppressors, leading to abnormal cellular proliferation and tumorigenesis. Its interaction with the NPC, particularly through nucleoporins like Nup214, highlights the intricate interplay between the transport machinery and the structural components of the NPC. Abnormal XPO1 activity allows excessive export of tumor suppressors from the nucleus, rendering them inactive and promoting cancer progression. Given this, targeting XPO1 with specific inhibitors presents a promising therapeutic strategy to restore normal transport dynamics, reduce oncogenic activity, and reinstate tumor-suppressor function. The continuation of the research into XPO1 inhibition extends the potential for developing effective cancer treatments by addressing both nuclear export dysfunction and its wider implications for nucleocytoplasmic transport regulation [13,34].

Additionally, recent studies have revealed that the NPC can function as a mechanosensor, changing its size, which is dependent on the nuclear envelope tension. This mechanism significantly impacts the molecular transport efficacy via nanopores [35]. Interestingly, Zimmerli et al. demonstrated that the energy levels and mechanical integrity of the nuclear membrane exert significant control over the diameter of NPCs, rendering them highly dynamic both spatially and temporally. This dynamic regulation encompasses exposure to mechanical stimuli such as cellular differentiation, migration, the transportation of large molecular cargoes, metastasis, and alterations in osmotic conditions. There are two main conformational statuses of NPCs distinguished: dilated and constricted. The functional implications of NPC dilation and constriction in active nuclear transport remain a subject for further investigation. However, it has been established that, during conditions of energy depletion and hyperosmotic shock, which induce a reduction in membrane tension, NPCs in Schizosaccharomyces pombe undergo constriction due to large-scale conformational changes arising from the movement of individual spoke complexes relative to each other [36,37].

## 2. Targeting Nuclear Pore Complexes

In this section, we will take a look at mechanisms relevant to nucleocytoplasmic transport in cancer biology and explore methods that can be used to target nuclear nanopores. Here, we list some key points to consider when evaluating the concept of targeting nuclear nanopores in cancer therapy. It is vital in the context of nuclear pore control to potentially stop cancer cells from proliferating, thus stopping tumor development.

Research in this area may hold promise for inhibiting nuclear nanopores via various mechanisms, such as disrupting the proteins involved in pore formation or transport. These mechanisms can be tailored to target specific types of cancer cells. These methods, among others, include mislocalization of proteins in subcellular compartments, inhibition of nuclear export, and activating oncogenes.

The number of nanopores is considered an important factor in nucleocytoplasmic transport capacity. As such, Tpr (NPC basket proteins) was found to regulate the total number of nuclear pore complexes per cell nucleus, which can result in increased nucleocytoplasmic transport capacity, providing multidrug resistance, as the cells with more Nups are more capable of exporting the drugs from the nuclei [38]. For instance, studies in cardiomyocytes and liver, brain, and cancer cells show a link between larger amounts of NPC presented and high proliferative capacity [39]. In cardiomyocytes with lower NPC numbers, there is less cell cycle activity, which was confirmed in a study by Han et al. in 2020 on neonatal mice with lower NPC numbers that exhibited less myocardial regeneration [40]. Sakuma et al., in 2021, also stated that inhibiting NPC formation leads to selective death of cancer cells, detained tumor growth, and even tumor regression [41]. All of the evidence listed above suggests that the formation of new NPCs could be restricted specifically to proliferative cells, making it a therapeutic target in cancer treatment [22].

### 2.1. Selectivity

Selectivity aims to minimize damage to healthy cells while targeting specific cancer cells, especially in tissues that are highly sensitive or potentially harmful if damaged, such as the brain or endocrine organs. Selectivity is partly mediated by nanopore diameter—it has been shown that pores smaller than 55 ± 5 nm in diameter show significant selectivity, which decreases as the diameter increases [42]. Interestingly, though, FG-binding molecules exhibit high-speed transport through the NPC, even when their molecular diameter significantly surpasses the exclusion limit for non-binding molecules, so binding can add up to a speed enhancement greater than the size of the particle [43].

### 2.2. Drug Resistance

Drug resistance is a major problem in cancer treatment due to the loss of DNA repair and cell cycle checkpoints [44]. A wide spectrum of cancers, such as CNS (Central Nervous System) cancers, including glioblastomas, are known for often developing resistance to treatments over time. It is important to consider the potential resistance mechanisms that cancer cells may develop against nuclear pore inhibition. Additionally, off-target effects and side effects may be associated with disrupting nuclear pores and need to be carefully studied and addressed in future research. An important mechanism underlying resistance is nuclear export [44]. Examples of strategies that can prevent nuclear transport include inhibitors of CRM1, antibodies targeting the nuclear export signal, and alterations to the structure of the nuclear pore complex (NPC). Such strategies may help reduce drug resistance in cancer cells or improve the ability to manage resistance mechanisms. The Nup62 protein, a key component of the NPC, has been studied in greater detail, and research has shown that its partial knockdown disrupts NPC composition and contributes to resistance to Cisplatin [45]. Additionally, increased Nup62 expression or staining has been linked to changes in NPC function, potentially impacting drug resistance.

### 2.3. Combination Therapies and Nanoparticles

Combining nuclear pore inhibition, specifically targeting either the structural components of the nuclear pore complex (NPC) or the inhibition of nuclear export via XPO1 (exportin 1), with other established cancer therapies such as chemotherapy or immunotherapy could enhance treatment outcomes. Identifying synergistic approaches, whether by disrupting the NPC structure to block transport or inhibiting XPO1 to prevent the export of tumor-suppressor proteins, is a critical aspect of ongoing cancer therapy research [46].

However, one should remember the potential disadvantages of combination therapies, such as the increased risk of unwanted side effects as a result of drug interaction. Another example of such a disadvantage is the possible opposite effects of medications used; for instance, one drug may inhibit the metabolic activity of the secondary one, which may lead to the buildup of overall treatment toxicity [47].

Combination treatments of 5-Fluorouracyl with topotecan or irinotecan have been proved to achieve high results in disrupting nuclear transport before disassembly of the NPC [48]. Their research reveals a new mechanism of action for these medications combined, creating even more potential therapeutic implications for using 5-Fluorourycal as an inhibitor of CRM1-induced nuclear export of tumor suppressors mediated by calcium. 5-Fluorouracil signaling through the calcium–calmodulin-dependent pathway is required for p53 activation and apoptosis in colon carcinoma cells [49].

Novel therapy approaches such as nucleus-targeting phototherapeutics should be considered in monotreatment and combination therapies as they demonstrate much higher anti-tumor effects compared to traditional DNA-targeting drugs [50]. It is important to notice that, although tissue penetration is greatly improved in this group of substances compared to visible light tissue penetration, it is still not a suitable option for a variety of deep-tissue tumors. The potential of nanoparticles in targeting NPCs has also been highlighted [51]. Tkachenko et al. demonstrated that gold nanoparticles (AuNPs) have potential in targeting nuclear pore complexes (NPCs) in HepG2 cancer cells. The authors used adenovirus RME and NLS for the modification of AuNPs, which allowed for selective drug delivery and nuclear targeting [52]. Another study highlighted that nanoparticles need to effectively bypass endosomal pathways and interact with the nuclear pore complex to facilitate transfer into the cell’s nucleus [53]. Skowicki et al. described supramolecular nanoassemblies, which are polymer- and peptide-based carriers that target the cell nucleus by exposing nuclear localization signals (NLS) to facilitate nuclear import. These nanosystems are designed to optimize treatment outcomes by increasing the concentration of therapeutic agents within the nucleus while minimizing off-target effects. The success of such delivery systems hinges on their ability to achieve cellular uptake, escape endosomal pathways, and interact with nuclear pore complexes (NPCs) to ensure precise nuclear translocation [54]. In another study, the authors developed a novel multifunctional nano-drug delivery system using mesoporous silica nanoparticles (MSNs) which were additionally modified with peptides for targeted cancer therapy and imaging. This system utilized HIV-1 TAT peptide for intranuclear delivery and YSA peptide to target the tumor-specific EphA2 receptor. This HIV-1 TAT peptide is known for its nuclear localization signal (NLS) and facilitates the translocation of the nanoparticles across the nuclear envelope by interacting with NPCs. Charge reversal through citraconic anhydride enabled controlled binding and release of the peptides in response to pH changes, allowing precise drug release and fluorescence recovery in acidic tumor environments. As was shown, this system is effective for drug delivery and tumor cell uptake, demonstrating equivalent inhibitory effects on MCF-7 cancer cells compared to free doxorubicin, making it a promising tool for combined cancer diagnosis and treatment [55]. As with any novel therapy, especially when dealing with cancers, ethical considerations around patient consent, safety, and informed decision-making must be prioritized in clinical trials and treatment development [56].

### 2.4. Potential Mechanisms

While targeting nuclear nanopores in cancer therapy is a promising avenue of research, it is important to recognize that it is likely a complex and evolving field that has yet to be explored. Further research, including pre-clinical and clinical studies, is needed to determine the feasibility and potency of this approach’s benefits. Moreover, it will be essential to carefully balance the therapeutic potential with the challenges and risks associated with disrupting nuclear pore function.

## 3. Defining NPCs as Potential Targets in Oncotherapy

**Figure 3:**
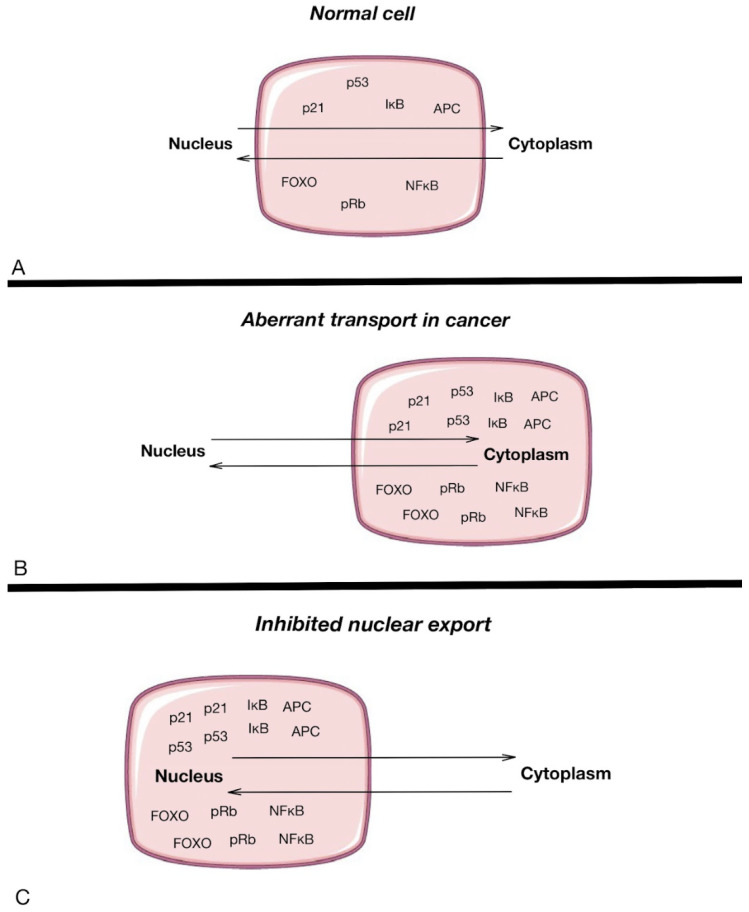
Nucleoplasmic transport in normal cells (**A**) and cancer cells (**B**), and the inhibition of nuclear export (**C**). The pink rectangle highlights the cell structure, with the upper section representing the nucleus and the lower section representing the cytoplasm, illustrating the location and movement of key proteins involved in nucleocytoplasmic transport. Arrows represent the movement of different proteins between the nucleus and the cytoplasm of a cell [13].

As previously mentioned, NPCs are the only turnstile embedded in the nuclear envelope, acting as nanoscale communicators between the cytoplasm and the nucleus (Figure 3). These structures regulate the transport of molecules, including essential regulatory proteins, between cellular compartments. Nucleoporins within the NPC play a critical role in controlling this transport, and alterations in these nucleoporins can lead to significant consequences in cancer progression. For instance, NUP98 fusions with other proteins have been implicated in leukemogenesis, while Nup214 and Nup358 are targets of chromosomal translocations that lead to hematopoietic malignancies. These structural changes in NPC components highlight their potential as therapeutic targets. However, the direct role of NPCs in carcinogenesis is still not fully understood. The studies of Rodriguez-Bravo et al. [57] identified elevated levels of NPC proteins such as POM121, Nup188, Nup210, Nup85, Nup62, and Nup214 in metastatic prostate cancer, indicating that NPC dysregulation may drive aggressive cancer phenotypes [58,59]. Interestingly, POM121 regulation was noted to be included in glycolysis in thyroid cancer, identified as a post-translational regulator of peroxisome proliferator-activated receptor (PPARΓ) and a factor that augments cell proliferation, migration, and invasion, thus making it a potential clinical therapy target and/or biomarker in colorectal cancer [60,61]. There are also some new data regarding Nups’ role in metastatic processes. An investigation was carried out by exploring the abilities of Nup210 [62]. Polymorphisms in the Nup210 promoter structure were found to affect CTCF binding, which, in turn, influenced Nup210 transcription. The expression of Nup210 has been linked to metastasis in human breast cancer patients, making it a potential metastasis susceptibility gene, particularly for ER+ breast cancers. Moreover, its depletion in mouse cancer cells caused decreased lung metastases. Additionally, Tpr was found to regulate the total number of nuclear pore complexes per cell nucleus, which can result in increased nucleocytoplasmic transport capacity, providing multidrug resistance, as cells with more Nups are more capable of exporting the drugs from the nuclei [38]. Another nucleoporin, Tpr, regulates the number of nuclear pore complexes per nucleus, which can increase nucleocytoplasmic transport capacity and contribute to multidrug resistance. Additionally, export defects may lead to the accumulation of cancer-promoting factors in the nucleus, driving cancer progression by preventing their proper localization in the cytoplasm [2].

## 4. Mislocalization of Transcription Factors Independent of NPC Defects

While NPC dysregulation plays a significant role in cancer progression, it is crucial to distinguish other factors contributing to the mislocalization of key regulatory proteins, such as transcription factors, that are not directly related to structural NPC defects. In many cases, the mislocalization of proteins, including p53, p21, and STAT proteins, is the result of dysregulated post-translational modifications, signaling pathways, or mutations in nuclear localization or export sequences. For example, the accumulation of wild-type p53 in the cytoplasm of solid tumors occurs not due to NPC defects but rather due to mechanisms that inhibit its nuclear import or enhance its cytoplasmic retention. This mislocalization prevents p53 from fulfilling its tumor-suppressive functions, contributing to cancer progression. Similarly, the mislocalization of p21 can shift its role from a tumor suppressor in the nucleus to a pro-survival factor in the cytoplasm. STAT3, an oncogene within individual cells, is transported through the NPC, but its dysregulation and oncogenic activity are typically driven by aberrant tyrosine phosphorylation and misregulation of upstream signaling pathways, not by structural changes in the NPC. STAT3’s activation and nuclear translocation play key roles in metastasis, angiogenesis, and immune evasion, with the potential for intervention at various steps in its signaling pathway. It is essential to acknowledge, however, that in certain types of cancer, STAT3 exhibits tumor-suppressive effects. Various steps in the path leading to STAT3 activation present potential targets for interference. These steps include tyrosine phosphorylation at Y705 by (oncogenic) tyrosine kinases, dimerization facilitated by phosphotyrosine/SH2 domain interactions, nuclear translocation, DNA binding to GAS (Γ-interferon activated sequence) elements, and interaction with cofactors [44,63]. A well-known tumor suppressor, p21, exhibits paradoxical behavior as it can promote tumor growth under certain cellular conditions. This dual role has been observed in connection with the HBx gene and the context of hepatocarcinogenesis. The molecular activities of p21 depend on its subcellular localization. When located in the nucleus, p21 may inhibit cell proliferation and promote apoptosis, whereas, in the cytoplasm, it may have oncogenic and anti-apoptotic functions. Notably, interferon has the capacity to keep p21 in the nucleus, representing one of the mechanisms underlying its anti-hepatocarcinogenic function. Moreover, forced expression of p21 induces the apoptotic response against cisplatin in glioma and ovarian cancer [64]. In these cases, the dysregulation of nucleocytoplasmic transport is not due to defects in the NPC structure itself but rather due to altered regulation of the transport machinery, post-translational modifications, or mutations that impact nuclear localization signals. This highlights the need for a separate therapeutic strategy aimed at correcting these regulatory imbalances in addition to targeting NPC structural defects.

## 5. Chemotherapy and NPC Inhibition

It was shown by Sakuma et al. in 2021 that inhibition of NPC formation leads to selective cancer cell death, preventing tumor growth and inducing its regression. This process is mainly restricted to cells in the proliferating stage [41]. In another piece of research, Ikliptikawati et al. showed that proteasomes, cellular structures responsible for protein breakdown, are located close to NPCs and that Nups play a crucial role in maintaining neural progenitor cells. Among these, Nup107 is identified as a potent oncogene in GBM (glioblastoma). Reduced Nup107 leads to the stabilization of the p53 protein in the cell nucleus. Nup153 is responsible for creating a platform for p53 degradation by tethering proteasomes. The PIM kinase mediates the degradation of p53 by blocking the proteasome pathway [65].

Moreover, the study shows that MDM2 is an essential mediator of p53 degradation, localized in the nucleus in U87 and A172 GBM cell lines, where p53 degradation occurs. Under low MDM2 levels, mono-ubiquitination of p53 occurs and leads to further modifications of p53, resulting in promoting MDM2 release and p53 export. Contrarily, under high MDM2 levels, poly-ubiquitination and degradation of p53 happen. Given the genomic amplification of MDM2 in GBM with wild-type TP53, MDM2 significantly promotes the degradation of p53. As a result, it is reasonable to suggest that cancer cells seldom encourage the export of p53 in an unstressed condition. Several studies have shown that changes specific to cancer in Nups play a role in the activity of cancer cells. Therefore, GBM cells need a high number of NPCs to structure the platform effectively for p53 degradation and mediate the transport through NPCs. In light of their findings illustrating the involvement of PIM kinase in constructing functional 26S proteasomes, using a PIM inhibitor with established anti-cancer drugs could offer therapeutic advantages for a subgroup of GBM patients characterized by high expression of MDM2 and wild-type p53 status [65].

Blocking the nuclear export of proteins, particularly the cancer drug target topo IIα, can be achieved through three potential approaches: (1) CRM1 inhibitors, (2) NES small molecule inhibitors, and (3) casein kinase 2 inhibitors, with the last preventing post-translational phosphorylation of topo IIα. The most commonly used method for direct nuclear export inhibition is the use of CRM1 inhibitors.

BRAF kinase plays a key role in driving the proliferation of metastatic melanoma, a highly aggressive tumor with a generally poor prognosis affecting over half of the patients diagnosed with this condition. Recent studies suggest a connection between BRAF activity and nuclear pore complexes (NPCs), indicating that alterations in NPC function may contribute to dysregulated nuclear-cytoplasmic transport in BRAF-mutant melanomas, further promoting tumor progression. It was shown that BRAF inhibitors improve the overall survival of metastatic melanoma patients by blocking cell growth signals with low toxicity to the healthy tissues. The downside of this treatment is the eventual development of resistance and, following that, recurrences or relapses that usually occur within a short time after BRAF inhibitor therapy.

CRM1 is also highly expressed in malignant melanoma and may be used as a negative prognostic indicator. Melanoma cells treated by CRM1 inhibitors, the most frequently used drugs to block the nuclear export of proteins, tend to go into G1 cell cycle arrest and demonstrate increased levels of wild-type p45 protein while decreasing levels of Survivin. However, complete and total tumor regression can be seen in combination therapy merging CRM1 and BRAF inhibitors together.

Leptomycin B, a secondary metabolite derived from Streptomyces bacteria, was the first identified CRM1 inhibitor. It works by alkylating a reactive cysteine residue known as cysteine 528 on CRM1, preventing its binding to the NES [66]. Consequently, it disrupts the export process by preventing the formation of the CRM1-NES-RanGTP complex. Introduction of Leptomycin B to humans in a phase I clinical trial resulted in dose-limiting toxicity with severe malaise and anorexia. A number of other CRM1 inhibitors have been developed, all sharing a common mechanism of action involving the inactivation of cysteine 528 (Table 2). Such medications are analogs of ratjadone, synthetic derivatives of Leptomycin B, and SINE (selective inhibitors of nuclear export).

Melphalan is an alkylating agent that induces DNA damage and results in cancer cell death; therefore, it is commonly used in the treatment of multiple myeloma. Doxorubicin is also known as a treatment of multiple myeloma. Tyrosine kinase and CRM1 inhibitors are used in leukemia patients.

Cisplatin is used by itself or in combination with other therapeutics in the treatment of advanced or relapsed cancers and cancers that cannot be treated with more traditional oncological treatments. Examples include bladder cancer, cervical cancer, malignant mesothelioma, non-small-cell lung cancer, ovarian cancer, squamous-cell carcinoma of the head and neck, neuroblastoma, and testicular cancer.

Proteasome inhibitors such as Bortezomib and carfilzomib are known to increase the expression of p53 and IkB levels in multiple myeloma cells [67].

The CRM1 regulates the cellular localization and function of numerous proteins that are crucial for the development of cancer. Extensive anti-cancer effects were observed in both pre-clinical studies and clinical trials (phases I–III) in solid tumors and hematological cancers. Among various proteins exported by CRM1, there are p53, FOXOs, p27, nucleophosmin, BCR–ABL, eIF4E, and Survivin, so inhibition of this export factor could affect multiple aspects of carcinogenesis. Such inhibition can result in the downregulation of cancer hallmarks, including genomic instability, sustained proliferation, resistance to cell death, and cellular energetics reprogramming, which explains its broad-spectrum anti-cancer activity. It is important to clarify that the term ‘broad spectrum’ in the context of anti-cancer activities does not imply ‘all spectrum’, so it does not indicate effectiveness against all types of cancers [68].

Originally discovered in yeast, the gene responsible for encoding CRM1 has been found to be crucial for maintaining the structural integrity of chromosomes at a higher order. In addition to its involvement in nuclear-cytosolic transport, CRM1 also contributes to centrosome duplication and spindle assembly, particularly in response to DNA damage. In terms of normal development, CRM1 is implicated in the following processes: regulating GNT (gastrula–neurula transition), overseeing larval progression, controlling centrosome duplication, and spindle assembly. Interaction of CRM1 with the BRCA1 gene (breast and ovarian cancer) is essential for a centrosome DNA damage checkpoint. CRM1 exclusively binds to the undimerized form of BRCA1, as the NES is an integral component of the binding domain involved in the heterodimerization of BRCA1 with its partner protein BARD1. The NES in both BRCA1 and BARD1 becomes masked upon the formation of the heterodimer. Based on these observations, a hypothesis proposes that CRM1’s ability to transport BRCA1, BARD1, and several BRCA1-BARD1 substrates to the centrosome increases the proximity between these proteins, which consequently promotes the formation of BRCA1-BARD1 dimers. This, in turn, catalyzes the ubiquitination of downstream substrates essential for regulating centrosome amplification during the DNA damage response [69]. XPO1 protein levels are elevated, among others, in PDAC (pancreatic ductal adenocarcinoma), which plays a role in blocking TSP (tumor-suppressing protein) function through constant nuclear export. Jiankun Gao et al. have shown that one of the SINE inhibitors, KPT-185, caused inhibition of cell growth, migration, and tumor invasion in PDAC and induced apoptosis and G2-M cell cycle arrest in the low nanomolar range (IC50s 150 nM) [70].

XPO1 interacts with Nup214 and Nup88 nucleoporins in the nuclear pore complex and exports cargo proteins containing NESs (nuclear export signals) from the nucleus. XPO1 protein controls cell proliferation in several mechanisms: the subcellular localization of NES-containing oncogenes and tumor-suppressor proteins, the control of the mitotic apparatus and chromosome segregation, and the maintenance of nuclear and chromosomal structures. The XPO1 protein level remains consistent throughout the cell cycle and is primarily localized to the nuclear envelope within specialized cellular structures known as CNoBs (CRM1 nucleolar bodies). CNoBs rely on RNA polymerase I activity, and it is suggested that they play a role in ribosome biogenesis. High levels of XPO1 protein are observed in ovarian carcinoma, glioma, osteosarcoma, pancreatic, cervical, and gastric cancers, are associated with high serum levels of ALP (alkaline phosphatase), and are correlated with increased tumor size, negative histological grade, and poor progression-free (PFS) and overall survival (OS) in human osteosarcoma.

In addition, increased XPO1 is known to correlate with elevated prognostic markers in pancreatic cancer, such as serum CEA and CA19.9, so it is theoretically possible to use it in predicting poor PFS and OS. Its higher levels also match up with high expression of phospho-serine10-p27, which is resistant to XPO1-mediated nuclear export, but reduced abundance of p27, leading to p27’s cytoplasmic localization and degradation. It may also be used as a prognostic factor in gastric cancer, where high XPO1 levels keep up with high serum CEA levels, more advanced tumor stages, positive Her2 status, and distant metastasis.

Hematological malignancies in which SINE compounds displayed the most memorable single-agent activity and provided a statistically significant survival advantage were models of non-Hodgkin lymphoma, chronic lymphocytic leukemia, acute myeloid leukemia, acute lymphocytic leukemia, and multiple myeloma. Selinexor, a selective inhibitor of exportin 1, additionally provided evidence of efficacy on its own in solid tumor xenografts including kidney, pancreas, prostate, breast, lung, melanoma, colon, gastric, and ovarian cancers, neuroblastoma, and sarcomas. Notable synergy was noted in selinexor used with diverse chemotherapies and targeted therapies, including platinum and taxanes, topoisomerase I and II inhibitors, dexamethasone, cytarabine, proteasome inhibitors, and a range of TKIs. Initial findings indicate that selinexor is generally well received, with the primary side effects being nausea, anorexia, and fatigue, which are predominantly of Grade I and II, reversible, and manageable with supportive care. Collectively, these findings suggest that selinexor can be safely administered over extended durations with side effects that are manageable to patients who have undergone extensive prior treatments and experienced relapse and/or refractory conditions. Selinexor exhibited individual efficacy not only in hematological but also in solid malignancies, demonstrating activity as a single agent in prostate, ovarian, cervical, and colorectal cancers. Moreover, it showed prolonged disease control in patients with head and neck cancer and sarcoma. Encouraged by the positive outcomes from phase I studies, several phase II studies of selinexor have been initiated in patients with AML, GBM, melanoma, prostate, ovarian, cervical, and endometrial malignancies [13].

Nuclear import is an analog process. The importins recognize NLSs through the various importinA (also called karyopherins) adapter proteins which heterodimerize with importinB1 subunits, able to interact with NPC, resulting in cargo A/B1 importin–trimer import into the nucleus. Various cancer cells exhibit the upregulated expression of importinB1 in order to facilitate tumor growth and progression. It was shown that inhibition and downregulation of importinB1 suppress the proliferation of cancer cells by limiting the entry of cancer-related transcription factors into the nucleus [71]. Ivermectin (IVM) was initially used as an anti-parasite medication and is now a crucial substance for studying nuclear import. It inhibits importinA and importinB1 through an effect on their heterodimer [72]. Ivermectin is a specific inhibitor of importin α/β-mediated nuclear import able to inhibit replication of HIV-1 and dengue virus. Recently, it has been shown that iVM, by regulation of several signaling pathways (mitochondrial respiration, angiogenesis, cancer stem cells (CSCs), PAH2-SID, P-Glycoprotein, Yes-Associated Protein 1 (YAP1), Wnt-TCF pathway responses, RNA helicase, KPNB1 protein, or Akt/mTOR pathway), prevents the development of various cancer types [73]. Interestingly, it was proven that the mitogen-activated protein kinase (MAPK)/extracellular-signal-regulated kinase 5 (ERK5) pathway, which is a target in many cancer therapies (increased expression reported in, e.g., melanoma), depends on importinA/B1 units, which opens new strategies for cancer therapy including IVM [74].

Survivin is a protein originally found in the cytoplasm crucial for the inhibition of apoptotic executors that, during apoptotic stress, relocate to the nucleus. Therefore, it acts as a sort of physiological switch to perform the apoptotic process. This relocation is due to the inefficient assembly of functional RanGTP–CRM1–Survivin export, which, in turn, is caused by apoptotic RanGTP gradient collapse and is supported by the NES domain in the Survivin protein. Interaction between CRM1 and RanGTP mediates such exclusion of Survivin from the nucleus [66]. One of the ways of regulating PTEN nuclear import is through neddylation, a post-translational modification where NEDD8, a small ubiquitin-like protein, is attached to target proteins, affecting their stability and function. Neddylation promotes tumor development, while nuclear PTEN is thought to exhibit tumor-suppressive functions. Neddylation is also known to contribute to the progression of breast cancer and is correlated with poor prognosis in these patients [75].

## 6. Conclusions

Nuclear pore complexes (NPCs) are crucial in controlling the access of transcription factors and other regulatory molecules to regulate transport-dependent gene expression. Dysregulation of nucleocytoplasmic transport significantly impacts cancer development and progression, as both structural defects in NPCs and broader transport pathway misregulations contribute to aberrant protein localization. In particular, the overactivity of exportin 1 (XPO1/CRM1) in various cancers, such as ovarian carcinoma, glioma, and pancreatic cancer, highlights the need to target both NPC function and nuclear export processes in therapeutic strategies. While NPCs facilitate selective molecular exchange, misregulation of key transport mediators like XPO1 leads to improper localization of oncogenes and tumor suppressors, further driving cancer progression. Effective cancer therapies may need to address both the structural integrity of NPCs and the regulatory pathways governing nucleocytoplasmic transport, including the inhibition of nuclear export via CRM1 inhibitors. Nanotherapy, with its precision, safety, and modifiability, offers significant advantages over traditional cancer treatments by enhancing targeted delivery and reducing drug resistance. Mesoporous nanoparticles, with their customizable properties, emerge as promising carriers for drug delivery. Although the use of NPC-targeted approaches in cancer therapy remains an emerging field, it presents promising prospects and potential benefits over conventional treatments, warranting further investigation and development. 

## Figures and Tables

**Table 2:** Most commonly used chemotherapy drugs that achieve better results when combined with direct CRM1 inhibitors, their function, and therapeutical indications.

Name of the Drug	Action in the Cell	Usage Indications	Reference
Melphalan	Increases DNA damage	Multiple myeloma	[67]
Cisplatin	Interferes with DNA replication	Advanced or relapsed cancers, cancers that cannot be treated with more traditional oncological treatments (bladder cancer, cervical cancer, malignant mesothelioma, non-small-cell lung cancer, ovarian cancer, squamous-cell carcinoma of the head and neck, neuroblastoma, and testicular cancer)	[67]
Melphalan	Increases DNA damage	Multiple myeloma	[67]
Bortezomib	Increases the expression of p53 and IkB levels	Multiple myeloma	[67]
Carfilzomib	Increases the expression of p53 and IkB levels	Multiple myeloma	[67]
Doxorubicin	Inhibits the progression of topoisomerase II, stops the process of replication	Multiple myeloma	[67]
